# Atrial fibroblast metabolic reprogramming in atrial fibrillation: a candidate mechanism within multicellular atrial cardiomyopathy

**DOI:** 10.3389/fcvm.2026.1948993

**Published:** 2026-09-28

**Authors:** Xiao-hai Jiang, Run-zhong Wang, Ya-wen Sheng, Ling Liu

**Affiliations:** Department of Cardiology, The Affiliated Taizhou People’s Hospital of Nanjing Medical University, Taizhou School of Clinical Medicine, Nanjing Medical University, Taizhou, Jiangsu, China

**Keywords:** atrial cardiomyopathy, atrial fibrillation, atrial fibrosis, cardiac fibroblasts, metabolic reprogramming

## Abstract

Atrial fibrillation (AF) commonly coexists with obesity, diabetes, and other cardiometabolic disorders. However, how systemic metabolic stress creates an arrhythmogenic atrial substrate remains unclear. This narrative review evaluates atrial fibroblast metabolic reprogramming as a testable mechanism within multicellular atrial cardiomyopathy, not as an established causal pathway. We organize the evidence by source: human atrial fibroblasts, human atrial tissue, atrial animal models, non-atrial cardiac fibroblasts, extracardiac fibroblasts, and reduced experimental systems. Studies of non-atrial cardiac and extracardiac fibroblasts independently implicate enhanced glycolysis, glutaminolysis, altered fatty acid metabolism, and redox or mechanosensitive signaling in myofibroblast activation. However, these processes were examined across different tissues and models and have not been shown to form a unified program in human atrial fibroblasts. Human atrial tissue and animal studies link metabolic stress, fibroblast activation, fibrosis, and AF but rarely establish fibroblast-specific metabolic causality. No study in the cited evidence set directly measured metabolism in human atrial fibroblasts; direct reports instead assessed activation, contraction, migration, and matrix output. We therefore position fibroblasts within a broader network of cardiomyocytes, endothelial cells, immune cells, adipocytes, smooth muscle cells, autonomic signaling, and thrombotic pathways. Therapeutic studies may support prevention or reverse atrial remodeling, but they do not show correction of human atrial fibroblast metabolism or regression of established atrial fibrosis. Direct cell-resolved profiling and atrium-specific, fibroblast-restricted perturbation are required to determine whether this mechanism is causal, reversible, and therapeutically actionable.

## 1 Introduction

Atrial fibrillation (AF) is the most common sustained cardiac arrhythmia, and its prevalence is rising with population aging and the growing cardiometabolic burden (1). It commonly coexists with obesity, type 2 diabetes, and metabolic syndrome. These comorbidities are associated with incident AF and recurrence after rhythm-control therapy. This overlap has shifted attention from an isolated electrical disorder to atrial cardiomyopathy (AtCM). AtCM encompasses structural, electrical, mechanical, functional, inflammatory, and prothrombotic remodeling. It can precede AF, whereas AF can accelerate the underlying atrial disease (2, 3). The relationship is therefore bidirectional and cannot be attributed to one cell type or pathway.

The broad clinical phenotype of AF further indicates that it should not be regarded as an isolated rhythm disorder. In an electronic health record–based study including 199,433 patients with new-onset AF and matched controls, Chung et al. found a substantial burden of cardiovascular, cerebrovascular, and other systemic conditions both before and after AF diagnosis.⁴ These findings underscore the clinical heterogeneity of AF and support a holistic management strategy integrating rhythm control with comorbidity management. Because this study primarily identified disease associations rather than causal relationships, its findings further suggest that AF should be understood beyond rhythm disturbance alone and in the context of its underlying atrial pathological substrate (4).

Among the structural components of this atrial substrate, interstitial and replacement fibrosis are particularly important because they disrupt myocyte coupling, slow conduction, increase conduction heterogeneity, and promote re-entry. Late gadolinium enhancement (LGE) on cardiac magnetic resonance imaging is associated with fibrosis burden and post-ablation recurrence (5). One LGE study found that fibrotic substrate extent predicted ablation outcome more strongly than pulmonary vein isolation alone (6). Fibroblasts and myofibroblasts are major matrix-producing effector cells. However, cardiomyocytes, endothelial and immune cells, adipose tissue, mechanical stress, and circulating signals shape their activity. Current rhythm-control therapies do not directly target these cellular or extracellular substrate components. This limitation motivates investigation of substrate-directed mechanisms without implying that fibroblasts alone determine clinical outcomes.

Experimental cardiomyocyte models and human atrial biopsies link AF to mitochondrial dysfunction. Altered mitochondrial calcium handling has also been demonstrated in experimental systems (7). Whether cardiomyocyte metabolic injury affects neighboring fibroblasts, or fibroblast metabolism mediates this crosstalk in the human atrium, remains unresolved.

Metabolic studies suggest that fibroblast-to-myofibroblast transition depends partly on changes in cellular energy and biosynthetic pathways. 6-Phosphofructo-2-kinase/fructose-2,6-bisphosphatase 3 (PFKFB3)-dependent glycolysis has been linked to profibrotic activation in non-atrial cardiac fibroblasts (8). Scleraxis-dependent glutaminase expression connects transforming growth factor-*β* (TGF-*β*) signaling to glutamine metabolism in cardiac fibroblasts (9). Separate studies implicate fatty acid metabolism, redox signaling, and matrix-stiffness sensing. Together, these findings make fibroblast metabolism a plausible contributor to activation. However, the evidence spans non-atrial cardiac fibroblasts, extracardiac tissues, animal models, and reduced systems. It therefore does not establish a coordinated program in human atrial fibroblasts.

This narrative review evaluates atrial fibroblast metabolic reprogramming as a testable link among cardiometabolic stress, atrial fibrosis, and AF. We distinguish direct atrial evidence from extrapolation using the framework in Table 1. Figure 1 provides the central synthesis, whereas Tables 2, 3 separate therapeutic evidence from clinical-context inference. We frame the proposition as a falsifiable candidate mechanism within multicellular AtCM, not as a completed causal chain.

**Figure 1:**
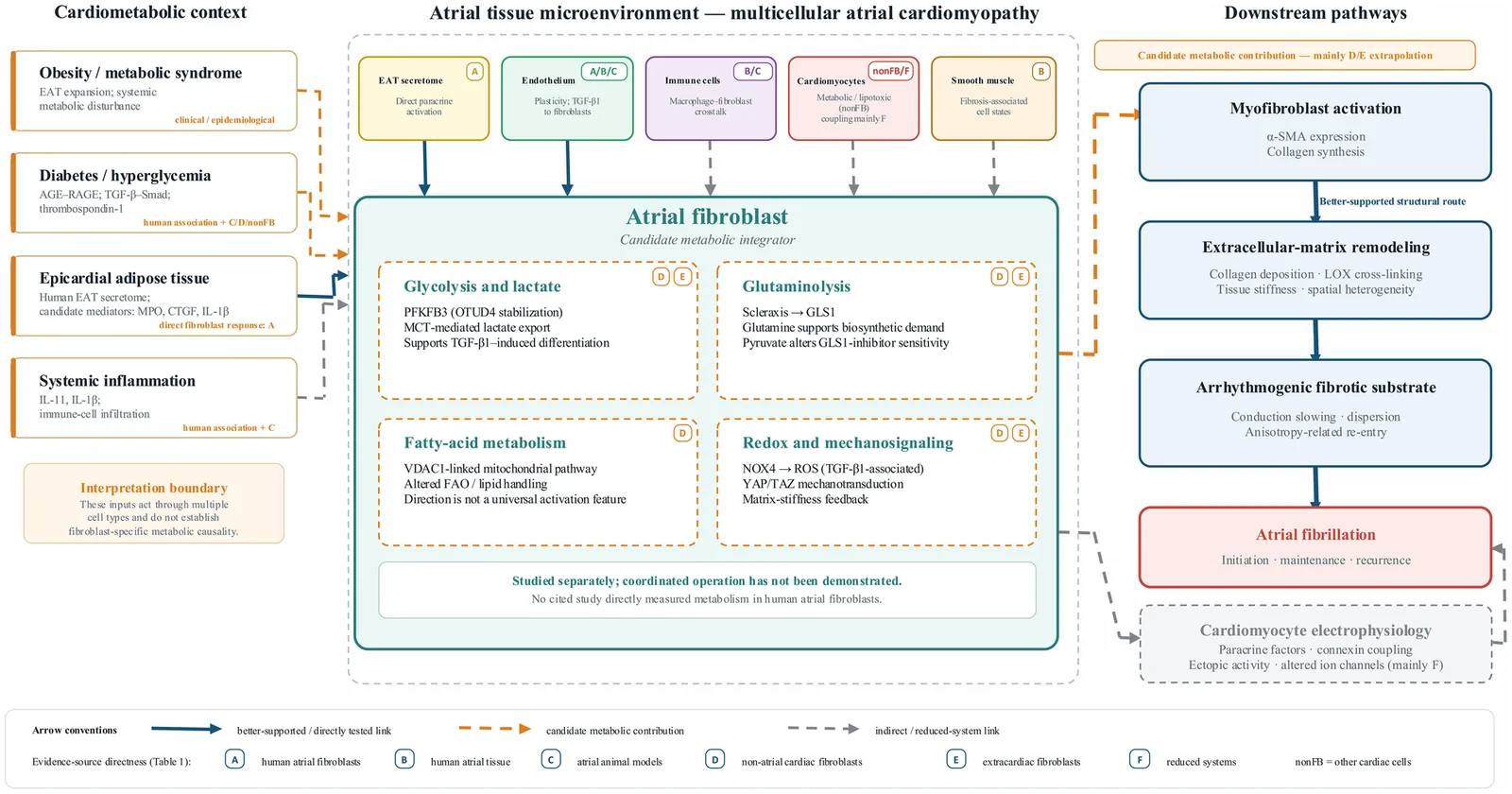
Proposed framework linking cardiometabolic stress, atrial fibroblast activation, and the arrhythmogenic substrate of atrial fibrillation. Cardiometabolic and multicellular signals may influence atrial fibroblasts through glycolytic, glutaminolytic, fatty-acid, and redox/mechanosensitive pathways, leading to myofibroblast activation, extracellular-matrix remodeling, and a fibrotic arrhythmogenic substrate. These metabolic pathways are supported largely by non-atrial or extracardiac evidence and have not been shown to operate as a coordinated program in human atrial fibroblasts. Solid, dashed orange, and dashed gray arrows indicate better-supported, candidate metabolic, and indirect/reduced-system links, respectively. Evidence categories A–F correspond to Table 1.

**Table 1:** Evidence-source directness framework for mechanistic statements about atrial fibroblast metabolism.

Level	Evidence source	Permitted inference	Principal limitation
A	Human atrial fibroblasts with cell-specific functional evidence	Direct support from isolated human atrial fibroblasts or fibroblast-resolved human atrial preparations when the relevant pathway or function is measured or perturbed.	Culture adaptation, sampling bias, loss of tissue context, or incomplete *in situ* validation may remain.
B	Human atrial tissue or descriptive cell-resolved atlases	Direct support for atrial pathology, association, spatial localization, or fibroblast state in human tissue without fibroblast-specific functional perturbation.	Bulk or histological data do not establish cell specificity; descriptive single-cell or single-nucleus data do not establish causality or function.
C	Atrial animal models	Supports atrial mechanisms and intervention effects *in vivo* or in animal atrial fibroblasts.	Species and model differences limit direct human inference.
D	Ventricular or general cardiac fibroblasts	Supports plausibility for cardiac fibroblast biology.	Requires chamber-specific extrapolation to the atrium.
E	Extracardiac fibroblasts	Supports a potentially conserved fibroblast mechanism across organs.	Requires organ- and disease-context extrapolation.
F	Reduced experimental systems	Supports mechanistic possibility in co-culture, engineered tissue, isolated coupling, or computational models.	Cannot establish quantitative importance in the intact human atrium.

Categories A–F indicate the directness of evidence for fibroblast-specific mechanistic inference, not methodological quality. Evidence outside the atrial/cell-specificity framework is not assigned a category. **nonFB**, mechanistic evidence from cardiac non-fibroblast cells.

**Table 2:** Therapeutic evidence matrix: clinical effect estimates, mechanistic directness, and inference boundaries.

Intervention or target	Clinical evidence by objective	Mechanistic evidence and directness	Defensible inference and remaining gap
SGLT2 inhibitors	Randomized-trial meta-analyses produced closely similar point estimates that differed in statistical significance: RR 0.888 (95% CI 0.806–0.978) in a network meta-analysis including standard-of-care comparators (59), RR 0.90 (0.80–1.01) in a placebo-controlled analysis (60), and RR 0.86 (0.77–0.96) in a larger placebo-controlled analysis of 52 trials, in which the effect was preserved in trials enrolling heart failure with reduced EF but not mildly reduced or preserved EF (*P* = 0.01) (61). AF was usually an adverse-event outcome, and the network meta-analysis found a significant reduction only for dapagliflozin (59). Post-ablation: a propensity-matched diabetes registry reported HR 0.63 (0.44–0.90); its study-level meta-analysis reported OR 0.61 (0.54–0.69), but treatment was not randomized (62). The DARE-AF randomized trial (*n* = 200), which excluded diabetes, heart failure, and chronic kidney disease, found no reduction in 3-month early recurrence (HR 1.11, 0.66–1.86) (63).	A (human atrial fibroblasts; non-metabolic endpoints): Empagliflozin attenuated transforming growth factor-β1-induced contraction and ECM remodeling in fibroblasts cultured from surgical atrial tissue (64) and reduced migration/procollagen I/III in commercial human atrial fibroblasts through NHE-linked pH/Ca^2+^ signaling (65). Neither study measured fibroblast metabolism. B (human atrial tissue): In right atrial appendages (*n* = 80; AF *n* = 18), inflammatory remodeling was associated with SGLT2 expression and *ex vivo* SGLT2 inhibition reduced ROS; immunofluorescence localized SGLT2 predominantly to endothelial cells and proinflammatory macrophages, with weaker cardiomyocyte colocalization, and no fibroblast marker was included (66). C (atrial animal models): Atrial animal studies report less fibrosis and, in diabetic models, lower ROS, conduction abnormalities, and inducibility (67–69).	Direct cultured-cell effects were measured on contraction, migration, and matrix output rather than metabolism, so they do not establish *in situ* correction of fibroblast metabolism. Human atrial tissue evidence is not fibroblast-specific and comes from a single manufacturer-supported study (66). Because DARE-AF excluded diabetes, heart failure, and chronic kidney disease, its neutral result does not test substrate modification in a metabolically loaded atrium. Clinical AF outcomes do not demonstrate regression of established human atrial fibrosis.
GLP-1 receptor agonists	Earlier randomized-trial meta-analysis: no overall AF association (70). Obesity-focused randomized-trial meta-analysis: incident AF RR 0.82 (95% CI 0.70–0.96; *I*^2^ = 0%) (71), an estimate for which possible data duplication has been raised in published correspondence (72). STEP-HFpEF was not powered for incident AF (73). Post-ablation meta-analysis, conservative random-effects estimate: HR 0.78 (0.61–0.99; *I*^2^ = 75%); a reconstructed individual-patient analysis in the same report gave HR 0.53 (0.38–0.72). Evidence mainly observational (74).	B (human atrial tissue): In the 2026 right-atrial-appendage study, inflammatory remodeling was associated with GLP-1 receptor expression and GLP-1 receptor modulation reduced ROS *ex vivo*; no fibroblast-specific mechanism was shown (66). Separate atrial-animal and non-atrial cardiac studies (C/D: atrial animal models and ventricular or general cardiac fibroblasts) report antifibrotic, electrophysiological, or cardiolipin effects (75–80).	No human atrial fibroblast target engagement has been shown. Clinical associations cannot be separated from weight, glycemic, hemodynamic, inflammatory, and heart-rate effects without prospective mechanistic trials.
Weight and risk-factor management	Structured weight/risk-factor programmes improved symptoms and structural measures in mixed AF populations (81, 82). Post-ablation SORT-AF primary endpoint: OR 1.14 (95% CI 0.37–3.61; neutral) (83). ARREST-AF: recurrent-arrhythmia HR 0.53 (0.32–0.89) with bundled management (84).	These interventions modify multiple systemic and paracrine inputs simultaneously. No category A (human atrial fibroblast) study has demonstrated metabolic correction in human atrial fibroblasts.	Supports context-specific risk-factor management, but weight loss alone and bundled management are not interchangeable. Neither proves regression of established interstitial fibrosis.
PFKFB3 and GLS1	No AF clinical efficacy studies identified in the cited evidence set.	Glycolysis and glutaminolysis are supported mainly in non-atrial cardiac or extracardiac fibroblasts (D/E) (8, 9, 20–23, 36).	Candidate experimental nodes only. Atrium-specific, fibroblast-restricted perturbation must show target engagement, fibrosis change, and AF change while monitoring metabolic compensation.
Fatty-acid metabolism, NOX4, and YAP/TAZ	No AF clinical efficacy studies identified in the cited evidence set.	Separate non-atrial cardiac and extracardiac studies (D/E) support individual roles in activation or mechanotransduction (26, 31–35).	Do not constitute a unified atrial fibroblast pathway. Systemic inhibition may affect multiple cell types; atrial delivery, safety, and reversibility remain unresolved.

Evidence categories are defined in Table 1. Clinical efficacy and mechanistic target engagement are evaluated separately; effect estimates are reported as published and should not be compared directly across heterogeneous designs or endpoints. AF, atrial fibrillation; CI, confidence interval; ECM, extracellular matrix; EF, ejection fraction; GLP-1RA, glucagon-like peptide-1 receptor agonist; GLS1, glutaminase 1; HR, hazard ratio; NHE, sodium–hydrogen exchanger; NOX4, NADPH oxidase 4; OR, odds ratio; PFKFB3, 6-phosphofructo-2-kinase/fructose-2,6-bisphosphatase 3; ROS, reactive oxygen species; RR, risk ratio; SGLT2, sodium–glucose cotransporter 2; TAZ, transcriptional coactivator with PDZ-binding motif; YAP, Yes-associated protein.

**Table 3:** Proposed clinical-context framework for evaluating metabolic and antifibrotic interventions in atrial fibrillation.

Clinical context	Primary objective and informative endpoint	What current evidence supports	Required mechanistic test or inference boundary
Cardiometabolic risk before documented AF	Prevent incident AF. Endpoint: adjudicated or monitor-detected first AF over a prespecified interval.	Four SGLT2-inhibitor meta-analyses reported fewer AF events (59, 61, 85, 86), whereas one placebo-controlled analysis found no statistically significant effect (60). In the largest placebo-controlled analysis the benefit was confined to trials enrolling heart failure with reduced ejection fraction (61). In most underlying trials, AF was captured as an adverse event rather than a prespecified efficacy endpoint. Epidemiological studies link obesity and diabetes to AF risk (37, 40).	Fewer AF events cannot establish substrate reversal. Pair clinical surveillance with serial atrial structure/function measures; fibroblast-specific biomarkers remain exploratory.
AF with limited measured substrate	Slow AF progression and reduce burden. Endpoints: continuous AF burden, transition to persistent AF, left atrial function and volume.	Cohort and trial data from mixed AF populations support improvements in symptoms and several remodeling measures, but these studies were not designed around this clinical context and did not demonstrate fibroblast target engagement (38, 81, 82). None of them measured atrial fibrosis directly, so their placement in this context is retrospective.	Define baseline substrate and repeat imaging/function measures. A change in atrial volume or symptoms should not be labeled fibrosis regression.
Progressive or persistent AF	Reduce AF burden and limit substrate progression. Endpoints: continuous burden, left atrial function, and quantitative fibrosis imaging.	An ovine obesity model with inducible AF supports reverse atrial remodeling after weight reduction (87), but it does not establish efficacy in persistent or progressive AF. Human evidence for regression of established interstitial fibrosis remains insufficient.	Stratify by baseline fibrosis and metabolic phenotype; test whether an intervention changes fibrosis independently of loading conditions and rhythm status.
Peri- or post-ablation	Reduce recurrence after the blanking period. Endpoints: continuous or protocolized rhythm monitoring, repeat ablation, cardioversion, and antiarrhythmic-drug use.	Randomized trials of weight and risk-factor management yielded different results: SORT-AF was neutral for its primary endpoint, whereas ARREST-AF showed lower recurrence (83, 84). SGLT2-inhibitor evidence is mixed: observational post-ablation associations were favorable, whereas DARE-AF was neutral for early recurrence (62, 63). GLP-1RA data are mainly observational or meta-analytic (74).	Recurrence reduction does not prove fibrosis regression. Measure substrate change and fibroblast target engagement separately from rhythm outcome.
Advanced/high-fibrosis substrate	Test reversal of established substrate. Endpoints: prespecified quantitative fibrosis reduction, functional recovery, AF burden, and durability.	Within the reviewed evidence, no intervention demonstrates both human atrial fibroblast target engagement and regression of established atrial fibrosis.	Require high baseline fibrosis, serial imaging with validated methods and, when clinically available, tissue analysis. Limited reversibility must remain an *a priori* possibility.

This framework is intended for evidence interpretation and is not a validated AF staging system or treatment algorithm. Clinical contexts may overlap. AF, atrial fibrillation; GLP-1RA, glucagon-like peptide-1 receptor agonist; SGLT2, sodium–glucose cotransporter 2.

## 2 Methods

This narrative review was informed by structured and targeted literature searches. PubMed and Europe PMC were searched through 16 August 2026 using combinations of terms related to atrial fibrillation, atrial cardiomyopathy, fibroblasts, fibrosis, metabolic pathways, and clinically relevant metabolic interventions. Additional studies were identified through targeted searches, reference-list screening, and publisher-site searches. Studies were selected when they addressed a prespecified evidence domain, irrespective of whether their findings supported or challenged the proposed mechanism. Primary mechanistic studies were prioritized, whereas reviews and consensus statements were used mainly for clinical context and definitions.

Mechanistic evidence was classified according to atrial and cell specificity using the framework in Table 1. These categories represent inferential directness rather than methodological quality. Key classifications were reviewed by all authors. Because this was a narrative rather than systematic review, no formal risk-of-bias assessment, PRISMA flow diagram, or quantitative synthesis was performed. The review should therefore be interpreted as a hypothesis-generating synthesis rather than an exhaustive evidence inventory.

## 3 The fibrotic substrate and the fibroblast as a matrix-producing effector

The electrophysiological effects of atrial fibrosis depend on extracellular matrix amount, composition, cross-linking, and spatial distribution, not on collagen accumulation alone. As major producers and organizers of this matrix, fibroblasts contribute to substrate formation through their activation.

Fibroblast-to-myofibroblast transition is commonly driven by TGF-β. Canonical Smad2/3 signaling promotes collagen synthesis, α-smooth muscle actin expression, and matrix-remodeling enzymes (10). Fibroblast-specific disruption of TGF-β-Smad2/3 signaling attenuates fibrosis in non-atrial cardiac injury models. This finding supports a causal role for fibroblasts in cardiac matrix deposition (11). Under Table 1, this constitutes category D evidence for cardiac fibroblast causality.

Fibroblast activation integrates biochemical and mechanical signals rather than responding to a single cytokine. Matrix rigidity favors myofibroblast differentiation, whereas TGF-β1 promotes differentiation across substrate rigidities in cultured human cardiac fibroblasts (12). This is category D evidence because the human cells were of unspecified chamber origin. Metabolic state may modulate activation alongside transcriptional, epigenetic, mechanical, and paracrine control.

Matrix amount, collagen isoform balance, covalent cross-linking, and spatial organization collectively affect tissue stiffness and conduction. Lysyl oxidase family enzymes catalyze collagen cross-linking and contribute to cardiac remodeling (13). Their induction after myocardial infarction is associated with mechanical dysfunction (14). These category D studies support a fibroblast-regulated matrix phenotype. However, direct evidence linking fibroblast-specific cross-linking to conduction in the intact human atrium remains limited.

Contemporary AtCM frameworks describe interactions among cardiomyocytes, fibroblasts, endothelial cells, smooth muscle cells, immune cells, and epicardial adipocytes. They also include autonomic and thrombotic pathways (2, 3, 15). Human single-cell studies identify smooth muscle and endothelial states associated with atrial fibrosis and region-specific endothelial states linked to thrombogenesis. These findings are category B observations (16, 17). Direct perturbation also places endothelial cells upstream of atrial fibrosis. One study combined single-cell analysis of human atrial tissue with lineage tracing and cell-restricted deletion in mice. Atrial endothelial cells showed mesenchymal activation with little complete conversion to fibroblasts. In isolated human primary atrial cells, endothelial-derived TGF-β1 promoted fibroblast proliferation and collagen production. Endothelial-specific Tgf-β1 deletion attenuated atrial fibrosis and reduced AF susceptibility after transverse aortic constriction (18). This study spans categories B, A, and C. Its mouse arm used an atrium-directed, cell-restricted perturbation with paired fibrosis and arrhythmia readouts. This design remains outstanding for fibroblast metabolism, as discussed in Section 8. Porcine and human data also show region-specific changes in fibroblast and macrophage populations in persistent AF (categories C and B) (19). Fibroblasts remain the dominant matrix-producing effector in this network, but not the dominant upstream controller of atrial disease. Current evidence provides the strongest cell-restricted causal demonstration for endothelial rather than fibroblast signaling.

Fibroblast activation can alter matrix quantity and material properties. Other cells and systemic signals independently affect electrophysiology, inflammation, vascular function, and thrombogenesis. Whether fibroblast metabolism acts as a modifiable integrator within this network, and under which disease conditions, remains unresolved.

## 4 Metabolic reprogramming of the activated atrial fibroblast

Fibroblast activation involves metabolic changes that may support proliferation and extracellular matrix synthesis. We consider four processes: enhanced glycolysis, glutaminolysis, altered fatty acid metabolism, and redox or mechanosensitive signaling. We review them together because each may meet the energetic or biosynthetic demands of activation. Available evidence does not show that they form one coordinated program. Direct evidence is insufficient to establish their simultaneous operation in human atrial fibroblasts.

Substantial experimental evidence supports glycolytic remodeling during myofibroblast differentiation outside the human atrium. In non-atrial cardiac fibroblasts, pharmacological or genetic PFKFB3 inhibition reduces TGF-β1-driven differentiation and fibrosis in cardiac injury models (20). OTUD4-mediated PFKFB3 stabilization also promotes fibroblast glycolysis and post-infarction fibrosis (8). In lung fibroblasts, PFKFB3-dependent glycolysis contributes to myofibroblast differentiation, including in cells from patients with idiopathic pulmonary fibrosis (21). These category D and E findings support a conserved profibrotic role for glycolysis.

Lactate handling provides related extracardiac evidence. In experimental pulmonary fibrosis, inhibiting monocarboxylate-mediated lactate export shifts fibroblast metabolism toward oxidative phosphorylation. It also limits TGF-β-driven differentiation and attenuates fibrosis (22). This category E evidence supports regulation of lung fibroblast state by lactate transport.

Glutaminolysis has independently been linked to the biosynthetic demands of cardiac fibroblast activation. Scleraxis induces glutaminase 1 (GLS1) in cardiac fibroblasts, connecting TGF-β-associated transcription to glutamine catabolism (9). In non-atrial heart-failure models, GLS1 inhibition reduces myofibroblast persistence and can improve fibrosis and cardiac function (23). This category D evidence supports a cardiac fibroblast mechanism but does not demonstrate simultaneous engagement with glycolysis.

Evidence for mechanical regulation of amino acid metabolism comes mainly from extracardiac systems. In tumor stroma, mechanical forces may link YAP/TAZ signaling to metabolic crosstalk (24). In systemic sclerosis, TGF-β1 is associated with increased glycolysis and glutamine metabolism. Inhibition of either pathway modifies profibrotic responses (25). These category E observations support mechano-metabolic coupling but not a common YAP/TAZ-GLS1-glycolysis axis in atrial fibroblasts.

A non-atrial cardiac fibrosis study linked fatty acid metabolism to fibroblast activation. Manipulation of voltage-dependent anion channel 1 (VDAC1) altered the fibrotic phenotype (26). This category D evidence suggests that mitochondrial lipid handling can influence cardiac fibroblast state. It does not establish suppressed fatty acid oxidation as a universal feature of atrial fibroblast activation.

Whether atrial fibroblasts share metabolic features with ventricular or extracardiac fibroblasts remains uncertain. In dogs, atrial fibroblasts were more reactive than ventricular fibroblasts, expanded their surface area faster, and expressed more α-smooth muscle actin. Platelet-derived growth factor signaling was also implicated (27). Separate atrial tissue and animal studies indicate sensitivity to metabolic disturbance. Peroxisome proliferator-activated receptor-γ coactivator-1α (PGC-1α) deficiency alters atrial expression of electrophysiological targets in mice (28). Human left atrial appendage proteomics also shows metabolic and myofibrillar changes in AF (29). These category B and C observations support an altered atrial environment.

Single-cell and single-nucleus studies are beginning to resolve the cellular sources of human atrial remodeling. Single-nucleus multi-omics identified three fibroblast subtypes in human left atrial appendages. The aFB2 state was enriched for fibrotic and smooth muscle contraction markers. By contrast, the NR4A1-marked aFB3 state was depleted for extracellular matrix reorganization gene sets and enriched for inflammatory pathways (30). Subtype proportions did not differ significantly between AF and sinus-rhythm appendages (30). NR4A1 knockdown in isolated human atrial fibroblasts reduced selected activation and extracellular matrix markers, although type I and III collagen expression was unchanged (30). This study provides category B evidence for *in situ* fibroblast states and category A evidence from isolated human atrial fibroblasts. However, it measured activation and matrix markers rather than metabolism. Smooth muscle cell states are also associated with atrial fibrosis (16). Endothelial signaling has been placed upstream of atrial fibrosis by cell-restricted perturbation (Section 3) (18). Region-specific endothelial states are also linked to thrombogenesis (17). None of these studies directly resolves the metabolic phenotype of fibroblast states *in situ*.

Cardiac and extracardiac studies independently link redox and mechanical signaling to fibroblast activation. NADPH oxidase 4 (NOX4)-derived reactive oxygen species contribute to TGF-β1-induced cardiac fibroblast differentiation (31). Matrix stiffness can also promote activation through YAP-associated mechanotransduction (32). Extracardiac and cross-organ fibrosis models further support YAP/TAZ-dependent activation (33–35). These category D and E studies used different systems. They therefore do not establish a single circuit connecting NOX4, mitochondrial redox signaling, YAP/TAZ, and metabolism in atrial fibroblasts.

Overall, the literature supports four separately observed metabolic or signaling processes, not a unified atrial fibroblast program. In primary human lung fibroblasts, pyruvate availability alters sensitivity to GLS1 inhibition (36). This category E evidence illustrates potential metabolic compensation and cautions against single-node inference.

PFKFB3, GLS1, fatty acid metabolic machinery, and NOX4/YAP/TAZ are therefore candidate experimental nodes supported mainly by category D or E evidence. They are not validated atrial therapeutic targets.

## 5 Upstream metabolic inputs that may influence atrial fibroblast reprogramming

The cardiometabolic milieu associated with diabetes, obesity, and epicardial adipose tissue (EAT) expansion may influence atrial fibroblast state. Evidence is most direct for high glucose and EAT-derived mediators tested in cardiac or atrial fibroblasts. By contrast, most lipotoxicity studies involve cardiomyocytes or whole myocardium. We therefore consider these pathways separately and do not assume convergence through fibroblast metabolism without direct testing.

In a real-world cohort of nearly 400,000 individuals, obesity was associated with increased AF risk (37). Goal-directed weight management in an AF cohort was associated with a dose-dependent reduction in arrhythmia burden (38). This pattern is compatible with atrial reverse remodeling. However, it cannot distinguish effects on fibrosis or fibroblast metabolism from hemodynamic, autonomic, inflammatory, or other systemic effects. EAT volume is associated with left atrial volume and atrial fibrosis in patients with AF (39). Diabetes is also associated with AF risk and adverse outcomes (40). These clinical and epidemiological observations lie outside the Table 1 framework. They motivate the biological question but do not identify a fibroblast-specific causal pathway.

The fibrillating atrium is itself metabolically remodeled, with changes in glucose, lipid, and ketone handling, mitochondrial function, and myofibrillar energetics (1). This bidirectionality complicates temporal and cellular attribution. The inputs below may act directly on fibroblasts or indirectly through an already remodeled atrial environment.

Interleukin-11 is elevated in patients with AF and correlates with circulating fibrosis markers. Its antagonism attenuates atrial fibrosis in angiotensin II-infused mice (41). This combination of human association and category C intervention evidence supports involvement in atrial fibrotic remodeling. It does not establish fibroblast-specific metabolic mediation.

High glucose increases extracellular matrix output in non-atrial cardiac fibroblast systems, with thrombospondin-1 and TGF-β1 implicated in collagen synthesis (42, 43). Advanced glycation end products (AGEs) can signal through their receptor (RAGE) and engage TGF-β-Smad pathways in cardiac fibroblasts (44). In diabetic rats, inhibition of AGE formation reduces connective-tissue growth factor expression and atrial interstitial fibrosis. This provides category C evidence for an atrial tissue effect (45). In diabetic mice, AGEs also affect atrial myocytes and AF susceptibility (46). These studies support an atrial role for glycation-related stress without identifying atrial fibroblast metabolic reprogramming as the causal intermediary.

Conditioned medium from human EAT induced fibrosis in an *ex vivo* rat atrial organ-culture model (47). This finding demonstrates profibrotic activity of the human EAT secretome but not *in vivo* causality in human atria. EAT secretome from patients with AF increased COL1A1 and FN1 expression in cultured human atrial fibroblasts. Proteomic and histological analyses identified myeloperoxidase as an abundant AF-associated component. However, the study did not show that myeloperoxidase mediated the fibroblast response (48). This constitutes category A evidence for a direct paracrine effect on human atrial fibroblasts, but not for a specific metabolic program. Connective tissue growth factor expression in human EAT is associated with AF and atrial fibrosis (49). EAT-derived interleukin-1β has also been implicated in postoperative AF (50). Together, these findings support EAT-fibroblast communication while leaving its metabolic intermediates and contribution to regional fibrosis incompletely defined.

Evidence linking lipotoxic intermediates to atrial fibroblast activation is indirect. In cardiomyocyte models, excess saturated fatty acids promote ceramide accumulation, mitochondrial dysfunction, oxidative stress, and cell death. Inhibiting ceramide synthesis prevents this lipotoxic phenotype (51, 52). These are nonFB studies under Table 1 because they examined cardiomyocytes rather than fibroblasts. They therefore cannot be ranked on the category A to F gradient. The evidence does not determine whether these lipids act directly on atrial fibroblasts or indirectly through stressed neighboring cells.

Oxidative stress may be common to hyperglycemic, inflammatory, and lipotoxic injury models. However, convergence of these pathways in the atrium has not been demonstrated (53). The dominant upstream input, relevant cellular redox compartment, and reversibility of established fibrosis remain unresolved.

## 6 From fibroblast activation to the arrhythmogenic substrate

The preceding evidence supports fibroblast activation and matrix production, whereas atrial fibroblast metabolic reprogramming remains a candidate upstream contributor. Structural, electrical, and paracrine routes differ markedly in evidentiary support. None establishes the complete metabolic-to-arrhythmia chain in humans.

Interstitial fibrosis can create collagenous barriers between myocyte bundles, slow conduction, increase spatial dispersion, and promote anisotropy-related re-entry (54). Matrix amount, architecture, and regional distribution may all affect conduction. These observations support a role for fibroblast-derived matrix in the arrhythmogenic substrate. They do not establish fibroblast metabolism as an upstream determinant in humans.

Evidence for direct electrical coupling comes mainly from co-culture and other reduced systems. In these models, myofibroblasts can couple to cardiomyocytes through connexins, depolarize adjacent cells, slow conduction, and promote ectopic activity (55, 56). Co-culture experiments and computational models suggest that arrhythmic effects vary with myofibroblast density (56, 57). This constitutes category F evidence for mechanistic plausibility.

Category F evidence indicates that cardiac fibroblast-derived factors can alter conduction and ion-channel expression in neonatal rat cardiomyocytes (58). Whether metabolically reprogrammed human atrial fibroblasts produce these signals at concentrations sufficient to influence AF remains unknown.

Most electrical evidence comes from reduced or non-atrial systems. Its contribution relative to the structural route in the intact human atrium therefore remains unknown. The *in vivo* interaction among structural, electrical, and paracrine routes is also an open quantitative question.

## 7 Therapeutic implications and the limits of mechanistic inference

Available evidence does not show that metabolic interventions modify the atrial substrate through fibroblast metabolism. Sodium-glucose cotransporter 2 (SGLT2) inhibitors, glucagon-like peptide-1 receptor agonists (GLP-1RAs), and weight loss affect many organs and cell types. Changes in AF events or atrial structure therefore cannot be assigned to fibroblasts without cell-specific measurements. Incident AF prevention, progression delay, post-ablation recurrence, and advanced-substrate reversal are distinct objectives. Table 2 separates clinical outcomes from fibroblast-specific target engagement. Table 3 defines the inference boundary across clinical contexts.

Meta-analyses of randomized trials yielded similar point estimates but differed in statistical significance. Three reported fewer AF events with SGLT2 inhibitors (59, 85, 86), whereas an updated placebo-controlled analysis found no statistically significant reduction (60). A larger placebo-controlled analysis of 52 trials reported a significant reduction (RR 0.86, 95% CI 0.77–0.96). The association persisted in trials of heart failure with reduced ejection fraction, but not mildly reduced or preserved ejection fraction (*P* = 0.01 for subgroup difference) (61). In the underlying trials, AF was generally recorded as an adverse event rather than a prespecified efficacy endpoint (59–61, 85, 86). The network meta-analysis found a significant reduction only for dapagliflozin (59). A Mendelian randomization study, outside the Table 1 framework, provides complementary but indirect evidence involving circulating lipoprotein traits (88). Direct experiments in human atrial fibroblasts show that empagliflozin attenuates TGF-*β*1-induced contraction and extracellular-matrix remodeling in cells from surgical atrial tissue (64). In commercially sourced human atrial fibroblasts, it also reduces migration and procollagen I/III production through NHE-linked pH and calcium signaling (65). These studies measured contraction, migration, and matrix output rather than metabolism. They do not establish metabolic correction *in situ* or regression of human atrial fibrosis.

A 2026 study examined 80 human right atrial appendages, including 18 from patients with AF. Inflammatory remodeling was associated with higher SGLT2 expression, and *ex vivo* SGLT2 inhibition reduced reactive oxygen species (66). Immunofluorescence localized SGLT2 mainly to endothelial cells and proinflammatory macrophages, with weaker cardiomyocyte colocalization. No fibroblast marker was examined, so fibroblast expression was neither demonstrated nor excluded (66). In hypertensive heart-failure rats, empagliflozin reduced both atrial and ventricular fibrosis (67). In diabetic rats, empagliflozin or dapagliflozin reduced atrial mitochondrial reactive oxygen species, fibrosis, conduction slowing, and tachyarrhythmia inducibility (68, 69). By contrast, the DARE-AF randomized trial found no reduction in 3-month early recurrence after ablation with dapagliflozin. Participants had persistent AF but not diabetes, heart failure, or chronic kidney disease (63). This neutral result therefore does not test substrate modification in a metabolically burdened atrium. A propensity-matched diabetes registry and study-level meta-analysis associated SGLT2-inhibitor use with lower post-ablation recurrence, but treatment was not randomized (62). These findings are divergent and do not establish substrate modification.

Experimental studies report less cardiac fibrosis after liraglutide and related GLP-1 signaling interventions in models of diabetes and other cardiac disorders (75–78). Liraglutide also modifies atrial electrophysiological remodeling in an animal model (79) and myocardial cardiolipin remodeling in obese mice (80). In the same 2026 human right-atrial-appendage study, GLP-1 receptor modulation reduced reactive oxygen species *ex vivo*. Combined SGLT2 and GLP-1 receptor modulation produced the largest reported effect, but no fibroblast-specific mechanism was identified (66). Together, these distinct models support the plausibility of cardiac and atrial effects.

Clinical findings for GLP-1RAs vary by population and endpoint. An earlier randomized-trial meta-analysis found no overall association between GLP-1RA use and AF (70). A STEP-HFpEF secondary analysis assessed semaglutide effects by baseline AF status. It was not designed or powered to test incident AF (73). By contrast, a later obesity-focused meta-analysis reported lower incident AF risk (RR 0.82, 95% CI 0.70–0.96; *I*^2^ = 0%) (71). Published correspondence has raised possible data duplication for this estimate (72). A 2026 post-ablation meta-analysis also reported lower recurrence. We cite its more conservative random-effects estimate (HR 0.78, 95% CI 0.61–0.99; *I*^2^ = 75%). The same report's reconstructed individual-patient analysis yielded HR 0.53 (0.38–0.72). The underlying evidence was mainly observational and methodologically heterogeneous (74). These findings justify prospective trials but do not identify the responsible pathway.

Weight-loss and lifestyle interventions support favorable atrial structural and electrophysiological remodeling. Histological evidence for reduced atrial fibrosis is limited to animal models. Regression of established human atrial fibrosis has not been demonstrated. In an ovine obesity model, weight reduction decreased atrial inflammation and fibrosis and improved electrophysiological substrate measures (87). In humans, structured weight and risk-factor management reduced AF symptoms (81). Cardiac magnetic resonance follow-up also showed lower left atrial volume and pericardial fat (82). Randomized post-ablation evidence is inconsistent. SORT-AF was neutral for AF burden (OR 1.14, 95% CI 0.37–3.61), whereas ARREST-AF reported less recurrent arrhythmia (HR 0.53, 95% CI 0.32–0.89) (83, 84). Weight loss alone and multifactorial management should therefore be considered distinct interventions.

Weight-loss and risk-factor programs simultaneously address blood pressure, glycemic control, sleep-disordered breathing, and cardiac adiposity (81, 82, 89). Rhythm or structural outcomes therefore cannot be attributed to a single fibroblast pathway. Even when rhythm or chamber measures improve, regression of established interstitial fibrosis and correction of fibroblast metabolism require separate demonstration.

Direct targeting of PFKFB3, GLS1, or NOX4 is mechanistically specific but remains preclinical for AF. As discussed in Section 4, these nodes are supported mainly by category D or E systems. None has corrected human atrial fibroblast metabolism or reversed atrial fibrosis in patients. Because these enzymes function in multiple cell types, systemic inhibition may cause off-target metabolic effects. Fibroblast-selective, atrium-directed delivery remains unresolved, and reversal of a fixed activated state is hypothetical.

Beyond the pathway-based targets discussed above, human genetic evidence offers a complementary strategy for therapeutic target prioritization and drug repurposing in AF. Kukendrarajah et al. identified 1,043 genes associated with AF or related traits, of which 68 had drug interactions encompassing 613 unique compounds. Their catalogue included the metabolism-related target SIRT1 and the inflammatory target IL6R, as well as the mitochondrial complex I-related genes NDUFAF3 and NDUFB10 linked to metformin. These findings are relevant to the metabolic and inflammatory pathways considered here, although the reviewed clinical evidence for metformin was observational and mixed. The prioritized proteasome target PSMB7 also connects this resource to protein homeostasis and inflammatory signaling; however, its associated inhibitors, including bortezomib and carfilzomib, carried pharmacovigilance signals of potential AF harm rather than established therapeutic benefit. Such findings can guide mechanistic testing, but neither genetic association nor a recorded drug-target interaction establishes the direction of a therapeutic effect or demonstrates correction of human atrial fibroblast metabolism. Cell-specific target engagement and effects on atrial fibrosis therefore require direct validation (90).

Clinical and experimental evidence supports context-specific hypotheses, not fibroblast-specific substrate modification. No intervention has demonstrated both target engagement in human atrial fibroblasts and regression of established fibrosis. Mechanistic studies and context-specific trials are therefore needed.

## 8 Conclusions and outlook

Atrial fibroblast metabolic reprogramming may link cardiometabolic stress to one component of the AF substrate, but the evidence is uneven. Individual processes are supported mainly by category D and E studies in non-atrial cardiac or extracardiac fibroblasts. Category B and C studies support metabolic and fibrotic remodeling in human atrial tissue and atrial animal models. However, no category A study in the cited evidence set measured metabolism in human atrial fibroblasts. Available category A reports assessed activation, contraction, migration, and matrix output instead. This proposed chain must therefore be interpreted within multicellular AtCM. Cardiomyocytes, endothelial cells, immune cells, adipocytes, smooth muscle cells, autonomic signaling, and thrombotic pathways may act in parallel or interact with fibroblasts.

Cross-organ conservation of glycolytic, glutaminolytic, redox, and mechanosensitive processes supports testable predictions, not atrial proof. We state two falsifiable predictions. First, the fibrotic aFB2 state identified by single-nucleus multi-omics in human left atrial appendages (30) should show greater glycolytic or glutaminolytic activity than the canonical aFB1 state. It may also show higher PFKFB3 or GLS1 protein. The absence of these differences across independent cohorts would argue against a glycolysis-glutaminolysis axis in the human atrium. Second, atrium-directed, fibroblast-restricted correction of one metabolic node should reduce atrial fibrosis and AF susceptibility *in vivo*. Metabolic flux should be monitored for compensatory rerouting (36). An endothelial-restricted version of this experiment has already been performed in an atrial animal model (18). The fibroblast-restricted equivalent is therefore technically feasible rather than aspirational (Section 3). Failure of either prediction would argue against the model.

The first priority is to define the metabolic phenotype of human atrial fibroblasts. Spatial metabolomics could be integrated with single-cell or single-nucleus transcriptomics, proteomics, validated fibroblast markers, and functional assays. Freshly isolated or *ex vivo* cells would be particularly informative. Human atrial studies show that bulk multi-omics and combined metabolomic-proteomic analyses are feasible. However, they lack spatial or fibroblast-specific metabolic resolution (91, 92). Lineage tracing and fibroblast-restricted perturbation are more appropriate for atrial animal models.

Second, causal attribution requires atrium-specific, fibroblast-restricted perturbation with measurement of both fibrosis and arrhythmia. Third, intervention timing should be tested across prevention, progression, post-ablation recurrence, and advanced substrate. Studies should also stratify participants by baseline fibrosis and metabolic phenotype. A plasma multi-omic study, outside the Table 1 framework, identified candidate circulating markers. Their prospective performance and relationship to atrial fibrosis or fibroblast state remain unverified (93). Table 3 aligns clinical context, baseline substrate, timing, endpoints, and mechanistic sampling without assuming that late fibrosis is reversible.

Current evidence therefore positions atrial fibroblast metabolic reprogramming as a candidate component of a multicellular substrate and a source of testable hypotheses. It is not an established master regulator or a validated therapeutic target.
